# Safe homecoming intervention after hospital discharge: a retrospective cohort study of mortality and readmission among patients 75 years or older in Sweden

**DOI:** 10.1186/s12877-026-08367-w

**Published:** 2026-10-06

**Authors:** Ulrika Westerling, Tahir Taj, Emma Nilsing Strid, Liselotte Hermansson, Mikko Hellgren

**Affiliations:** 1 University Health Care Research Centre, Faculty of Medicine and Health, Örebro University, Örebro, Sweden; 2 Clinical Epidemiology and Biostatistics, School of Medical Sciences, Faculty of Medicine and Health, Örebro University, Örebro, Sweden; 3 School of Medical Sciences, Faculty of Medicine and Health, Örebro University, Örebro, Sweden; 4 Kumla Primary Health Care Centre, Örebro County Council, Örebro, Sweden

**Keywords:** Frail older adults, Continuity of patient care, Comprehensive health care, Primary health care, Transitional care, Community health services

## Abstract

**Background:**

After a hospital stay, older patients are at increased risk of health complications that may lead to readmission to hospital or premature death. Globally, numerous interventions have been applied to enhance transitional care for older patients, but the evidence base remains inconclusive. One form of a transitional care intervention is the Swedish primary health care *Safe homecoming* intervention, a time-limited, interprofessional and tailored home health care intervention designed to prevent avoidable complications and readmissions following hospital discharge. The effects of *Safe homecoming* have not yet been scientifically evaluated. The present study aimed to assess the association between the interprofessional *Safe homecoming* intervention and the risk of death or readmission.

**Methods:**

We conducted a retrospective propensity score-matched cohort study in Örebro County, Sweden, using administrative and medical-record data from 2013 to 2020. The source cohort comprised 38,338 patients aged 75 years or older who had been admitted to hospital for at least one day. We included 128 patients who received the interprofessional *Safe homecoming* intervention at a single primary health care centre between 1 November 2016 and 31 May 2019 and 512 propensity score-matched controls from across the county receiving standard care.

**Results:**

The mean age was 85.5 years, and 60.9% of patients in both groups were women. At 90 days, the cumulative incidence of first hospital readmission was 20.3% in the intervention group and 33.6% among controls (Gray’s test *p* = 0.005). In the competing-risk analysis, the Fine-Gray subdistribution hazard ratio was 0.57 (95% CI 0.37–0.86; *p* = 0.008). The corresponding cause-specific Cox model yielded an HR of 0.55 (95% CI 0.36–0.84; *p* = 0.005). At 365 days, the Fine-Gray estimate was 0.79 (95% CI 0.59–1.05; *p* = 0.098). Mortality estimates favoured the intervention group numerically but were imprecise, with confidence intervals including the null at all evaluated follow-up times.

**Conclusion:**

*The Safe Homecoming* intervention was associated with a lower risk of first hospital readmission at 90 days, whereas there was no clear evidence of an association with mortality or longer-term readmission. Given the observational design, small intervention sample, potential residual confounding and evaluation of multiple follow-up time points, these findings should be considered exploratory and require confirmation in larger prospective studies.

**Supplementary Information:**

The online version contains supplementary material available at https://doi.org/10.1186/s12877-026-08367-w.

## Background

Older patients, especially those who are frail, face an increased risk of complications and hospital readmission after discharge [1–3]. In addition to age and frailty, length of hospital stay also affects the risk of readmission and premature death [4, 5]. Various interventions have been introduced globally to improve discharge care and reduce the risk of readmission [6–8]. These interventions, commonly referred to as “transitional care interventions” which aim to provide continuity of care at discharge, may improve health outcomes for older patients and make an important contribution to care delivery [6–8]. However, the complexity and heterogeneity in study designs, follow-up time, outcome measures, care components and services make it difficult to compare and evaluate the results of these interventions [6, 8, 9]. To optimise transitional care and avoid readmissions, a comprehensive approach that addresses multimorbidity, frailty, geriatric syndromes and polypharmacy is suggested [2, 3, 10, 11]. Despite the need to include geriatric aspects, research focusing on transitional care interventions for frail older patients is scarce. The effectiveness in improving health outcomes ought to be clearly established and further evidence is needed [6, 9, 10, 12].

In Sweden, primary health care is divided between regional and municipal providers, posing risks for delayed post-hospital care. To address this, several municipalities have implemented the *Safe homecoming* intervention [13]. While the standard *Safe homecoming* model is delivered by municipal assistant nurses in collaboration with standard homecare services [14], one municipality in Örebro County further developed the model to include a general practitioner and a physiotherapist, creating an interprofessional *Safe homecoming* model. This interprofessional model is designed to support older patients with complex care needs by promoting health, safety and home-based care, while reducing mortality and readmission risks. Family members are involved, where appropriate, to further strengthen continuity and support during the transition from hospital to home. Despite its widespread use in Swedish municipalities, the *Safe homecoming* intervention has not been scientifically evaluated. More research is crucial to build evidence-based transitional care for older patients.

The aim of this study was to evaluate whether receipt of the interprofessional *Safe homecoming* intervention, involving primary care and municipal home care staff, was associated with the risk of death or readmission among patients aged 75 years or older, compared with matched patients receiving standard care.

The primary hypothesis was that the all-cause mortality rate would decrease among patients up to 12 months after they had received the intervention. The secondary hypothesis was that all-cause readmissions would decrease among patients up to 12 months after receiving the intervention.

## Methods

### Design

This is a retrospective propensity score-matched cohort study [15] conducted in Örebro County, Sweden, based on administrative and medical records.

### Setting

During the study period, with data collected between 2013 and 2020, Örebro county had three public hospitals (including the University Hospital of Örebro) and 28 primary health care centres. The county had a mean population of 298,000, 9.8% of whom were 75 or older, distributed over twelve self-governed municipalities. One of these, Kumla, where the intervention was conducted, had a population of 22,000, with 9.0% of inhabitants aged 75 or older, similar to the national proportion of 9.3% [16]. The socioeconomic structure in Kumla is considered representative for Örebro county [17]. During the study period no other setting in the county had access to the *Safe homecoming* intervention. In this article, the term *Safe homecoming* refers to the interprofessional model including a general practitioner and a physiotherapist in addition to the standard team, unless otherwise specified.

### Study cohort

**Fig. 1:**
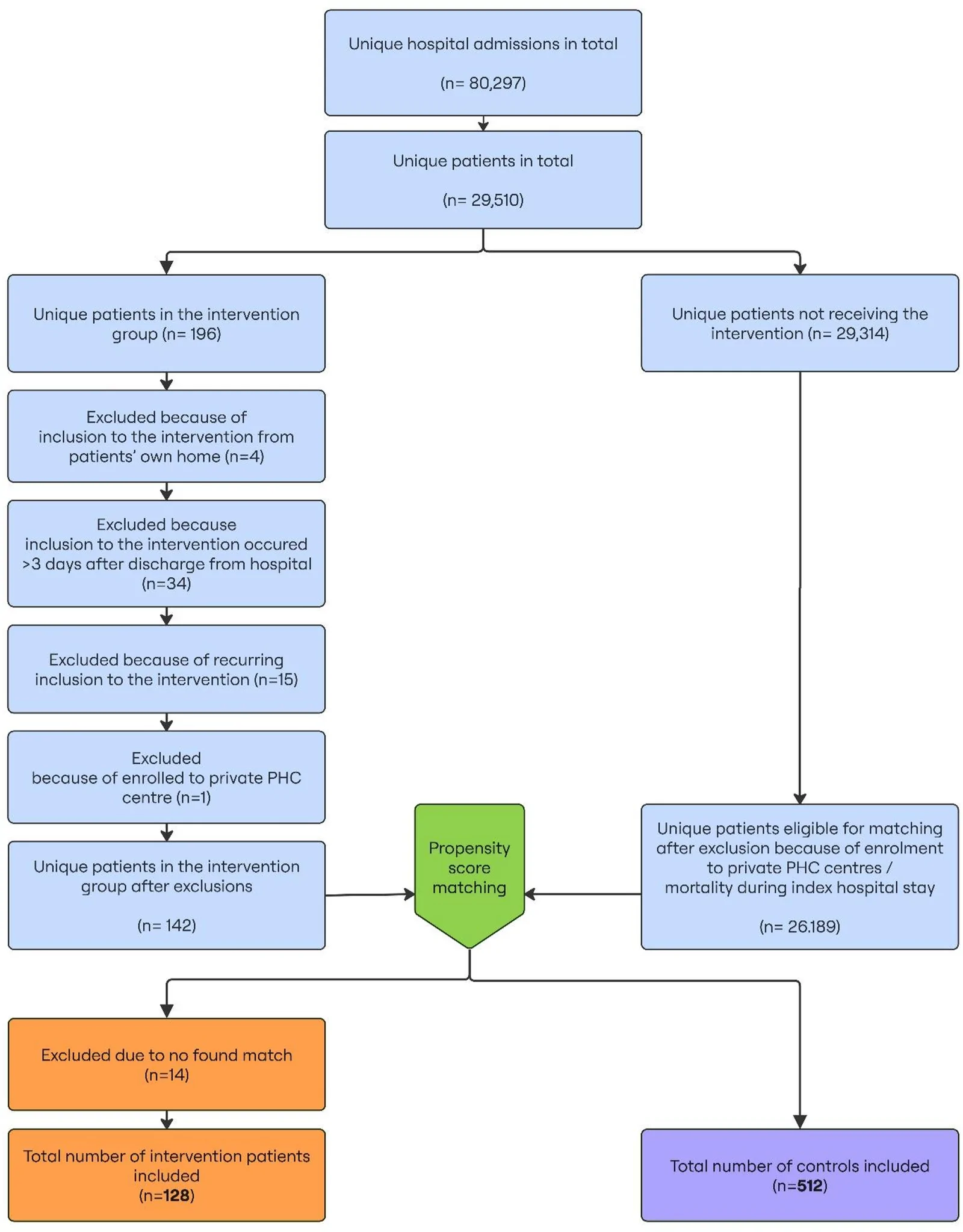
Flowchart of inclusion to the study. PHC, primary health care

The cohort comprised 38,338 patients aged ≥ 75 years who had been admitted for at least 1 day to one of the three hospitals in Örebro county. During the intervention period 2016–2019, 196 patients received the *Safe homecoming* intervention. After application of the study eligibility criteria, 128 patients were included in the intervention group. The selection process and reasons for exclusion are shown in Fig. 1. Patients in the intervention group were included if they received the intervention within 3 days of hospital discharge. While inclusion criteria were defined for the study, enrolment into the *Safe homecoming* intervention occurred in routine practice. Upon notification from hospital staff, the general practitioner at the Kumla primary health care centre and registered nurses from Kumla municipality involved with the *Safe homecoming* intervention assessed patient eligibility for inclusion, based on anticipated need for medical and social support immediately post-discharge. The control group consisted of propensity score matched [18] patients who received standard care. Matching was based on age, sex, weighted Charlson Comorbidity Index (CCIw), number of prescribed medications, duration of the index hospital stay, and main diagnosis category according to the International Classification of Diseases, 10th revision (ICD-10) at baseline; CCIw, medication count and hospital-stay duration were entered as categorical variables (for details, see Supplementary Table 1, Additional file 1). Exclusion criteria applied to both the intervention and the control group were: patients ≥ 75 years admitted to a psychiatric ward, patients enrolled to private primary health care centres because of no access to Anatomical Therapeutic Chemical (ATC) codes [19], and patients who died during a hospital stay at baseline. Patients in the intervention group were excluded if they: received the intervention without a preceding hospital admission, received the intervention > 3 days after hospital discharge, had multiple entries into the intervention (only the first intervention episode was retained for analysis) or lacked a suitable matched control (Fig. 1). Altogether 128 patients who received the *Safe homecoming* intervention in Kumla municipality and 512 matched controls from across Örebro County were included, see Fig. 1. Controls were selected from hospital discharge occurring between 2014 and 2020 whose inclusion date differed by no more than two years.

The index hospital discharge date was defined as baseline for outcome follow-up in both intervention patients and matched controls. *Safe Homecoming* was initiated on the day of discharge for all included intervention patients except one, for whom initiation occurred one day after a Sunday discharge; outcome follow-up for this patient also started on the hospital discharge date. Controls were selected from eligible hospital discharges between 2014 and 2020, with the control index date differing from that of the corresponding intervention patient by no more than two years. Matching was performed without replacement, and each intervention patient was matched to four controls.

### Intervention

**Fig. 2:**
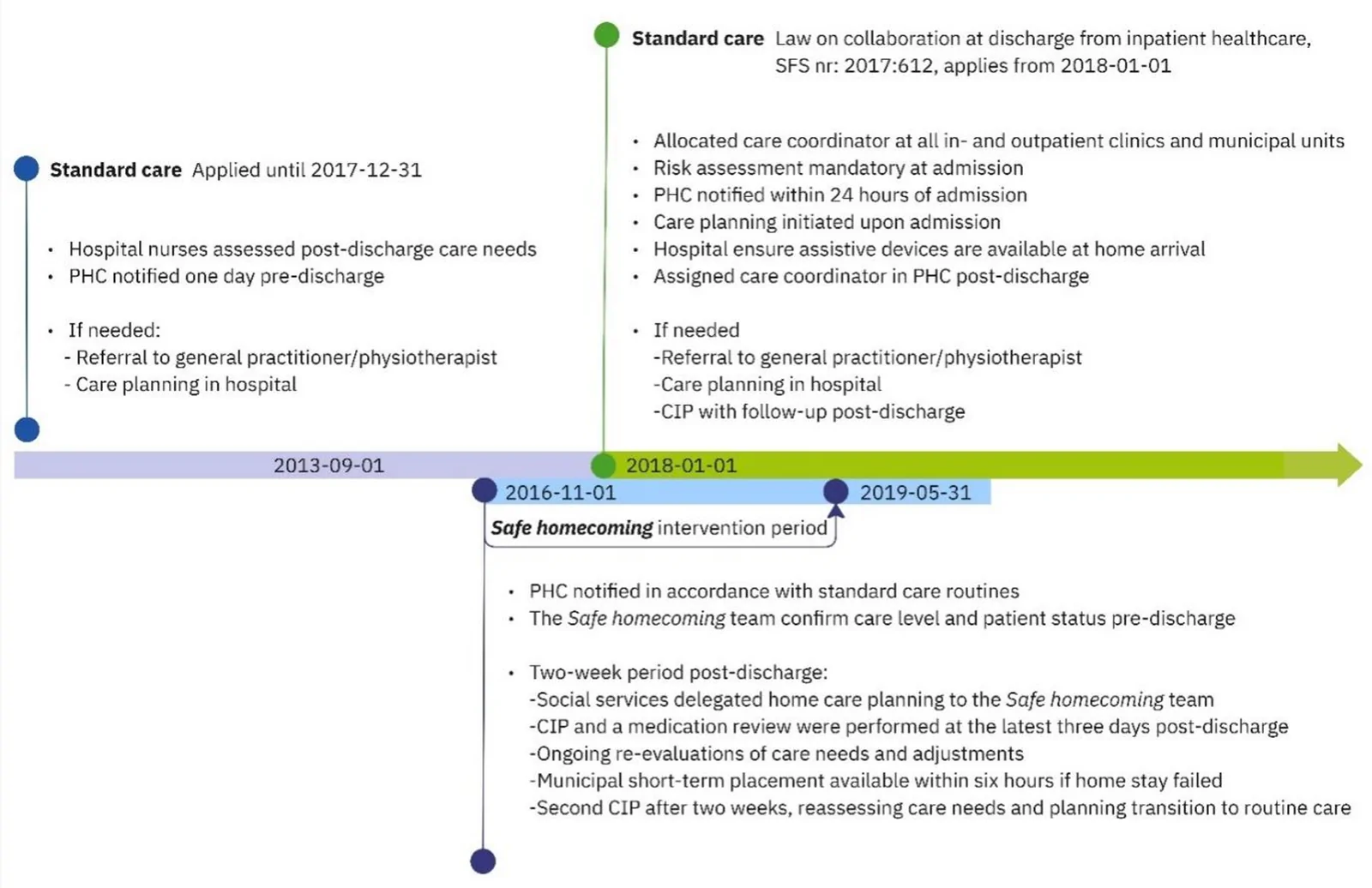
Main activities in planning for hospital discharge in the *Safe homecoming* intervention vs. standard care. CIP, coordinated individual planning; PHC, primary health care

The intervention was available from 1 November 2016 to 31 May 2019 and was delivered by the *Safe homecoming* team comprising a general practitioner and a physiotherapist from the primary health care centre, together with municipal registered nurses, assistant nurses, and an occupational therapist. Key components of the intervention are summarised in Box 1. Following notification of hospital discharge, through established standard care routines (Fig. 2), the *Safe homecoming* team assessed patients’ suitability for the intervention, and eligible patients were offered participation. The intervention aimed to enable patients with substantial care needs to be discharged directly to their own homes rather than to a municipal short-term care facility. Prior to discharge, the general practitioner reviewed the intervention patient’s medical records and medications, and where appropriate, liaised with hospital physicians regarding the patient’s current condition, ongoing treatment, follow-up needs and discharge plans. Care planning was predominantly undertaken in intervention patients’ homes, although some care planning was undertaken prior to discharge, enabling assessment of functional status in everyday environment and promoting active participation in care-related decision-making. Following discharge, all intervention patients received a joint home visit from the *Safe homecoming* team on the day of discharge (or the next working day for weekend discharges), including a medical assessment comprising physical examination, medication reconciliation, discussion of treatment plans, and where appropriate, initiation of dose-dispensed medications. During the initial home visit, an individualised and coordinated care plan was developed, with family members involved in the planning process when applicable. Registered nurses and occupational therapist were delegated authority to make decisions regarding social care services during the first 14 days following discharge. This arrangement aimed to facilitate timely provision of medical, rehabilitative, and social support. Personal alarm systems were installed when required. The overall aim was to maximise intervention patients’ independence, self-sufficiency and sense of safety.

The *Safe homecoming* team provided intensive support during the first two weeks after discharge. Assistant nurses delivered most daily care, while registered nurses, occupational therapist, physiotherapist and general practitioner contributed profession-specific assessments and interventions according to intervention patient’s needs. The team continuously reassessed intervention patient’s conditions and adjusted medical treatment, rehabilitation and care accordingly. Regular communication was maintained through telephone contact between the general practitioner and the other team members, supplemented by structured weekly interprofessional case discussions held both at the primary health care centre and in patients’ homes, as well as ad hoc general practitioner home visits when needed. The team operated during office hours. Patients in need of care outside office hours were supported by the regular home care and home health care services. However, if the intervention patient could not stay at home, a short-term municipal placement was arranged within 6 h. Approximately 14 days after hospital discharge, a second coordinated individual care planning meeting was conducted in the intervention patients’ home, additionally involving a municipal care manager. Responsibility was then usually transferred to regular home care and home health care services, although *Safe homecoming* support could be extended when necessary.

### Standard care

During the study period, for which data were collected between 2013 and 2020, two different routines for discharge planning were applied (Fig. 2). Until 31 December 2017 [20], hospital nurses, together with social workers, were responsible for conducting care planning prior to discharge when a patient required continued care. In Örebro county, a collaborative model was introduced in autumn 2013 with the aim of improving the quality and conditions for integrated care [21]. Despite the model, representatives from primary health care rarely participated in the planning process. Following the introduction of new legislation in 2018 [20], the main responsibility for ensuring continuity of care was transferred from hospitals to the regional primary health care services (see Fig. 2).

Box 1. Key components of the *Safe homecoming* intervention.

- Interdisciplinary team-based care.
- Direct discharge to home.
- Pre-discharge medical review and care coordination.
- Home-based assessment and individualised care planning.
- Coordinated home visit immediately after discharge.
- Intensive transitional support for 14 days, or longer if needed.
- Integrated health and social care.
- Promotion of independence, participation, and safety.

### Data collection, variables and study outcomes

Data were collected from administrative and medical records from 2013 to 2020. The data included the patients’ age, sex, primary health care provider, hospital unit, dates of hospital admission and discharge, discharge location, date of mortality, all inpatient and outpatient diagnoses (ICD-10 codes), outpatient medication (ATC codes), prescription dates and the prescribing primary health care centre. Data included information on the 12-month period before baseline to assess morbidity using CCI scores and 12 months after baseline to evaluate outcome. Baseline data involving age, sex and propensity score matched covariates were analysed at group level.

Length of hospital stay was measured in days; transfer between wards was counted as one care occasion. ICD-10 codes recorded during the year preceding baseline and at the index hospital admission were used to calculate the CCI scores based on a coding algorithm adapted for register-based research in Sweden [22, 23]. The CCIw score points were categorised as 0; 1–2; 3–5; ≥6 [24]. Prescribed medications were categorised using the ATC classification system [19], with first-level therapeutic groups employed to indicate the corresponding organ systems; over-the-counter drugs were not included. To categorise the number of medications, we applied established definitions: no polypharmacy (zero to four medications), polypharmacy (five to nine medications) and extensive polypharmacy (ten or more medications) [25]. Readmission was defined as hospital admission to any of the three hospitals in Örebro County within 365 days after baseline. Although municipal register data regarding municipal care services (e.g. short -term nursing home care, home care services, and other municipal-managed services) were not available, intervention -specific data were accessible and used to identify patients who had received the *Safe homecoming* intervention.

#### Outcome measures

The primary outcome was all-cause mortality within 365 days of discharge. The secondary outcome was time to first all-cause readmission within 365 days of discharge.

Crude cumulative mortality and readmission were summarised at 7, 14, 30, 60, 90, 180 and 365 days. Kaplan–Meier analyses were used to describe overall survival and readmission-free survival [26, 27]. Model-based analyses were performed with follow-up administratively censored at 30, 90, 180 and 365 days. For conventional time-to-first-readmission analyses, death before readmission was treated as a censoring event.

#### Sample size and statistical analysis

All eligible intervention patients (*n* = 128) with matched controls (*n* = 512) were included. No power calculation was performed. The statistical analysis was planned and performed together with an epidemiologist (TT) and the data analysis plan was established prior to the analyses. Although patients were followed individually, data were analysed at a group level.

Propensity scores were estimated using logistic regression, with treatment status as the dependent variable and the covariates as predictors (for details, see Supplementary Table 1, Additional file 1). We performed nearest-neighbour matching without replacement, aiming for a 1:4 ratio of intervention patients to controls, using a calliper of 0.2 standard deviations of the logit of the propensity score. In practice, this 1:4 matching ratio was achieved for all interventions: each treated patient was matched with four controls (128 intervention patients and 512 controls; total *n* = 640). The covariate balance was assessed using standardised mean differences, with values ≤ 0.1 indicating good balance. For categorical variables, the maximum level-specific standardised mean differences was used. Sensitivity analyses included a Love plot showing absolute standardized mean differences for each covariate before and after matching (for details, see Supplementary Fig. 1, Additional file 1).

Covariate balance after propensity score matching was evaluated using standardised mean differences (SMDs), rather than hypothesis-testing p-values, with an absolute SMD ≤ 0.10 considered indicative of acceptable balance for the variables included in the propensity-score model. Detailed balance diagnostics before and after matching are presented in Supplementary Fig. 1, Additional file 1).

Kaplan–Meier methods were used to estimate overall survival and readmission-free survival. Differences between groups were evaluated using log-rank tests. Cox proportional hazards regression was used to estimate hazard ratios (HRs) for mortality and first hospital readmission. For readmission models, death before readmission was treated as a censoring event; these models therefore estimate cause-specific hazards. Robust sandwich standard errors clustered by propensity-score matched subclass were used to account for dependence within matched sets. Separate Cox models were fitted with follow-up administratively censored at 30, 90, 180 and 365 days.

Because death precludes subsequent hospital readmission, competing-risk analyses were additionally performed for first readmission. Cumulative incidence functions (CIFs) were estimated with death before readmission treated as a competing event and compared between groups using Gray’s test. Fine–Gray regression was used to estimate subdistribution hazard ratios (sHRs), with robust standard errors clustered by propensity-score matched subclass. The Fine–Gray model estimates the association between the intervention and the cumulative incidence of readmission in the presence of the competing risk of death, whereas the cause-specific Cox model estimates the instantaneous hazard of readmission among patients who remain alive and readmission-free.

In five patients, readmission and death were recorded on the same calendar day. Because exact within-day ordering was unavailable, these observations were classified as readmissions in the primary competing-risk analysis. A sensitivity analysis classifying same-day events as deaths yielded materially similar results.

The proportional hazards assumption for the Cox models was assessed using scaled Schoenfeld residuals, with no evidence of violation. Effect estimates are presented with 95% confidence intervals. Because multiple outcomes and follow-up horizons were evaluated without adjustment for multiplicity, findings at individual follow-up times were interpreted cautiously. Statistical analyses were performed using R version 4.3.3 and SPSS version 29 (IBM Corp, Armonk, NY, USA).

## Results

### Baseline characteristics

Propensity score matching improved balance in the variables included in the matching model. Post-matching balance was assessed using standardised mean differences, with detailed balance diagnostics presented in Supplementary Fig. 1, Additional file 1. Baseline characteristics and additional descriptive diagnostic information are presented in Table 1 and Supplementary Tables 2 and 3, Additional file 1. Patients’ mean age was 85.4 years in the intervention and 85.6 years in the control group and women represented 60.9% of each group. Distribution of hospital stay categories, CCIw categories and medication categories was nearly identical between groups. The distribution of main diagnosis categories at baseline likewise showed good balance. Diagnostic patterns in both groups reflected high morbidity; hip fractures and stroke dominated in the intervention group, while stroke, fractures and heart failure were most common in the controls. Pneumonia occurred equally between the groups (see Supplementary Tables 2 and 3, Additional file 1). The study population’s baseline characteristics are presented in Table 1.

**Table 1:** Baseline characteristics of the intervention and control groups using propensity score matching

Variables	Intervention patients (*n* = 128	Matched controls (*n* = 512)
Sex, male/female, %	39.1/60.9%	39.1/60.9%
Age, yrs, mean (SD)	85.4 (5.7)	85.6 (5.2)
Age category, 75–80 years*	33 (25.8%)	112 (21.9%)
Age category, 81–85 years*	26 (20.3%)	130 (25.4%)
Age category, 86–90 years*	46 (35.9%)	185 (36.1%)
Age category, 91–95 years*	18 (14.1%)	64 (12.5%)
Age category, ≥ 96 years*	5 (3.9%)	21 (4.1%)
CCIw score at inclusion, mean (SD)	1.46 (1.59)	1.43 (1.59
CCIw score at inclusion, min–max (median)	0–7 (1)	0–9 (1)
CCIw category at inclusion, 0 (%)*	43 (33.6%)	170 (33.2%)
CCIw category at inclusion, 1–2 (%)*	61 (47.7%)	244 (47.7%)
CCIw category at inclusion, 3–5 (%)*	20 (15.6%)	80 (15.6%)
CCIw category at inclusion, ≥ 6 (%)*	4 (3.1%)	18 (3.5%)
Hospital stay, 1–6 days	28 (21.9%)	112 (21.9%)
Hospital stay, 7–14 days	67 (52.3%)	268 (52.3%)
Hospital stay, ≥ 15 days	33 (25.8%)	132 (25.8%)
ATC medications, 0–4*	37 (28.9%)	148 (28.9%)
ATC medications, 5–9*	44 (34.4%)	178 (34.8%)
ATC medications, ≥ 10*	47 (36.7%)	186 (36.3%)
All other ICD-10 code categories at baseline excluding S&T, n %*	95 (74.2%)	417 (81.4%)
ICD-10 code category, S&T, n %*	33 (25.8)	95 (18.6%)

ATC, Anatomical Therapeutic Chemical classification; CCIw, weighted Charlson Comorbidity Index; ICD-10, International Classification of Diseases, 10th revision; ICD-10 code category, "S&T" codes prefixed with S or T (injury, poisoning and certain other consequences of external causes); SD, standard deviation

*Data are presented as n (%) for categorical variables and mean (SD) for continuous variables unless otherwise specified

### Overall outcomes

Among the 640 matched patients, 182 (28.4%) died and 323 (50.5%) experienced at least one hospital readmission during the 365-day follow-up. Table 2 presents cumulative event counts and proportions from baseline through each specified follow-up time. At 90 days, 26 of 128 intervention patients (20.3%) and 172 of 512 controls (33.6%) had experienced a first hospital readmission (*p* = 0.005). At 365 days, the corresponding proportions were 44.5% and 52.0%. Mortality estimates were numerically lower in the intervention group across the reported follow-up times, but between-group differences were uncertain. Table 3 presents Kaplan-Meier estimates of overall survival and readmission-free survival across follow-up.

**Table 2:** Crude cumulative all-cause mortality and first hospital readmission at specified follow-up times

Time point	Crude mortality rates			Crude readmission rates		
	Intervention (*n* = 128)	Control (*n* = 512)	*P*-value	Intervention (*n* = 128)	Control (*n* = 512)	*P*-value
7 days	0 (0.0%)	13 (2.5%)	0.082	6 (4.7%)	39 (7.6%)	0.334
14 days	1 (0.8%)	21 (4.1%)	0.098	11 (8.6%)	63 (12.3%)	0.308
30 days	6 (4.7%)	43 (8.4%)	0.220	16 (12.5%)	99 (19.3%)	0.094
60 days	10 (7.8%)	68 (13.3%)	0.123	24 (18.8%)	140 (27.3%)	0.060
90 days	15 (11.7%)	87 (17.0%)	0.186	26 (20.3%)	172 (33.6%)	0.005
180 days	21 (16.4%)	117 (22.9%)	0.143	45 (35.2%)	209 (40.8%)	0.284
365 days	30 (23.4%)	152 (29.7%)	0.196	57 (44.5%)	266 (52.0%)	0.161

Values are cumulative numbers and percentages from the index hospital discharge through each specified follow-up time. Percentages were calculated using the total number of patients in each treatment group as the denominator. P-values were calculated using the chi-square test or Fisher’s exact test when appropriate.

**Table 3:** Kaplan–Meier estimates of overall survival and readmission-free survival

Time point	Overall survival		Readmission-free survival	
	Intervention (*n* = 128) % (95% CI)	Control (*n* = 512) % (95% CI)	Intervention (*n* = 128) % (95% CI)	Control (*n* = 512) % (95% CI)
7 days	100 (N/A)	97.5 (96.1–98.8)	95.3 (91.7–99.0)	92.4 (90.1–94.7)
14 days	99.2 (97.7–100.0)	95.9 (94.2–97.6)	91.4 (86.7–96.4)	87.7 (84.9–90.6)
30 days	95.3 (91.7–99.0)	91.6 (89.2–94.0)	87.5 (82.0–93.4)	80.7 (77.3–84.2)
60 days	92.2 (87.7–97.0)	86.7 (83.8–89.7)	81.2 (74.8–88.3)	72.7 (68.9–76.6)
90 days	88.3 (82.9–94.0)	83.0 (79.8–86.3)	79.7 (73.0–87.0)	66.4 (62.4–70.6)
180 days	83.6 (77.4–90.3)	77.1 (73.6–80.9)	64.8 (57.1–73.7)	59.2 (55.1–63.6)
365 days	76.6 (69.6–84.3)	70.3 (66.5–74.4)	55.5 (47.5–64.8)	48.0 (43.9–52.6)

CI, confidence interval; N/A, not applicable. Values are Kaplan–Meier probabilities with 95% CIs. In the readmission-free survival analysis, death before first readmission was treated as a censoring event

### Standard survival analysis of time to death

**Fig. 3:**
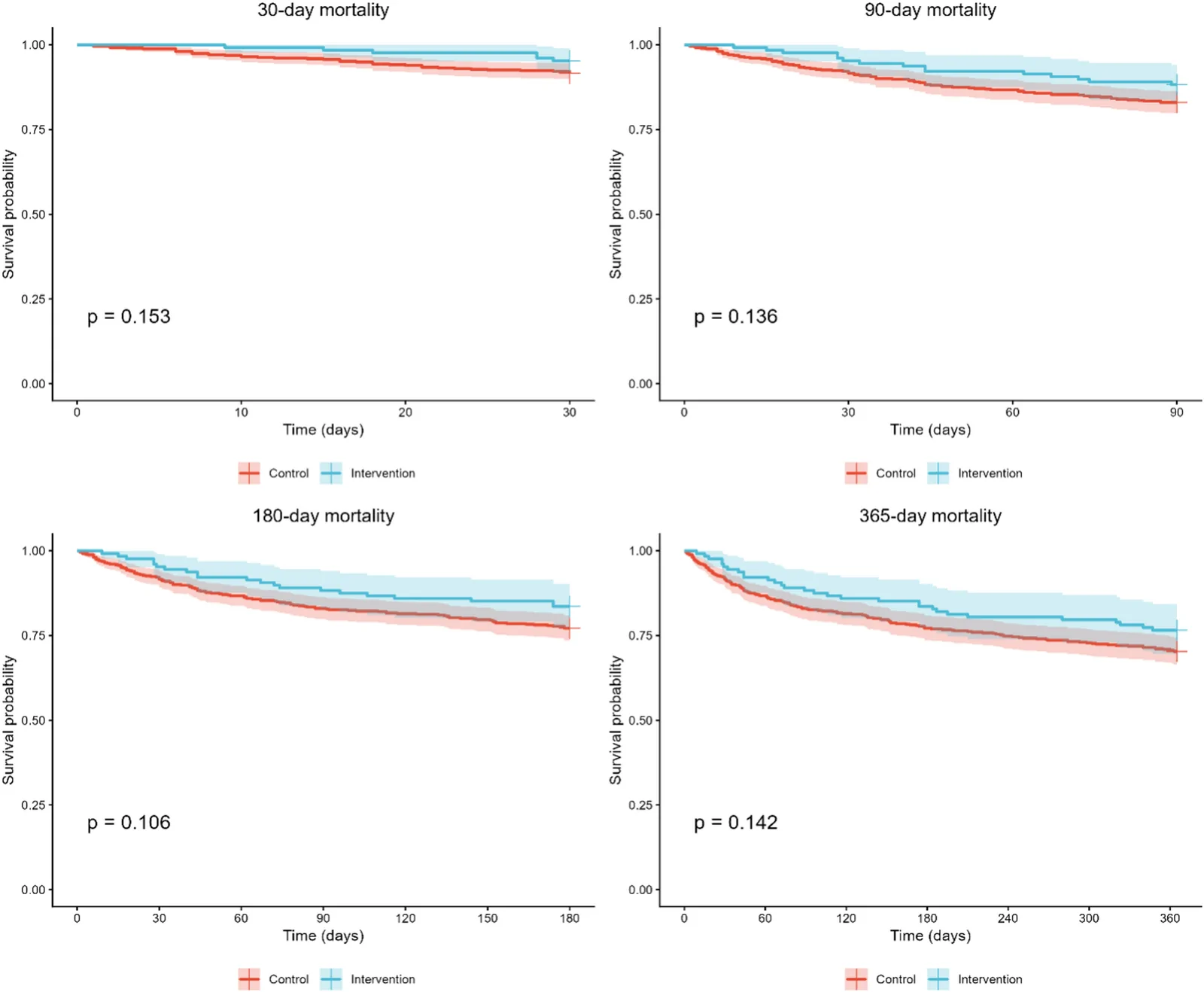
Kaplan-Meier survival estimates of mortality at 30, 90, 180 and 365 days

Kaplan–Meier estimates of overall survival were numerically higher in the intervention group throughout follow-up (Fig. 3). Consistent with the Cox analyses, however, the differences were uncertain. The robust Cox models produced mortality HRs below 1 at each evaluated follow-up horizon, but all 95% confidence intervals included 1 (Table 4).

**Table 4:** Cox proportional hazards models, all-cause mortality and first hospital readmission, intervention patients versus matched controls

Time point	Mortality HR (95%CI)	*P*-value	First readmission HR (95%CI)	*P*-value
30 days	0.54 (0.23–1.29)	0.167	0.61 (0.35–1.05)	0.076
90 days	0.66 (0.39–1.13)	0.130	0.55 (0.36–0.84)	0.005
180 days	0.68 (0.43–1.08)	0.104	0.76 (0.57–1.03)	0.078
365 days	0.75 (0.52–1.07)	0.110	0.75 (0.57–0.99)	0.042

CI, confidence interval; HR, hazard ratio. Controls were the reference group (HR = 1.0). Cox models used robust sandwich standard errors clustered by propensity-score matched subclass. In readmission models, death before first readmission was treated as a censoring event; the resulting HRs therefore represent cause-specific hazards. The proportional hazards assumption was assessed using scaled Schoenfeld residuals, with no evidence of violation.

### Standard survival analysis time to readmission

**Fig. 4:**
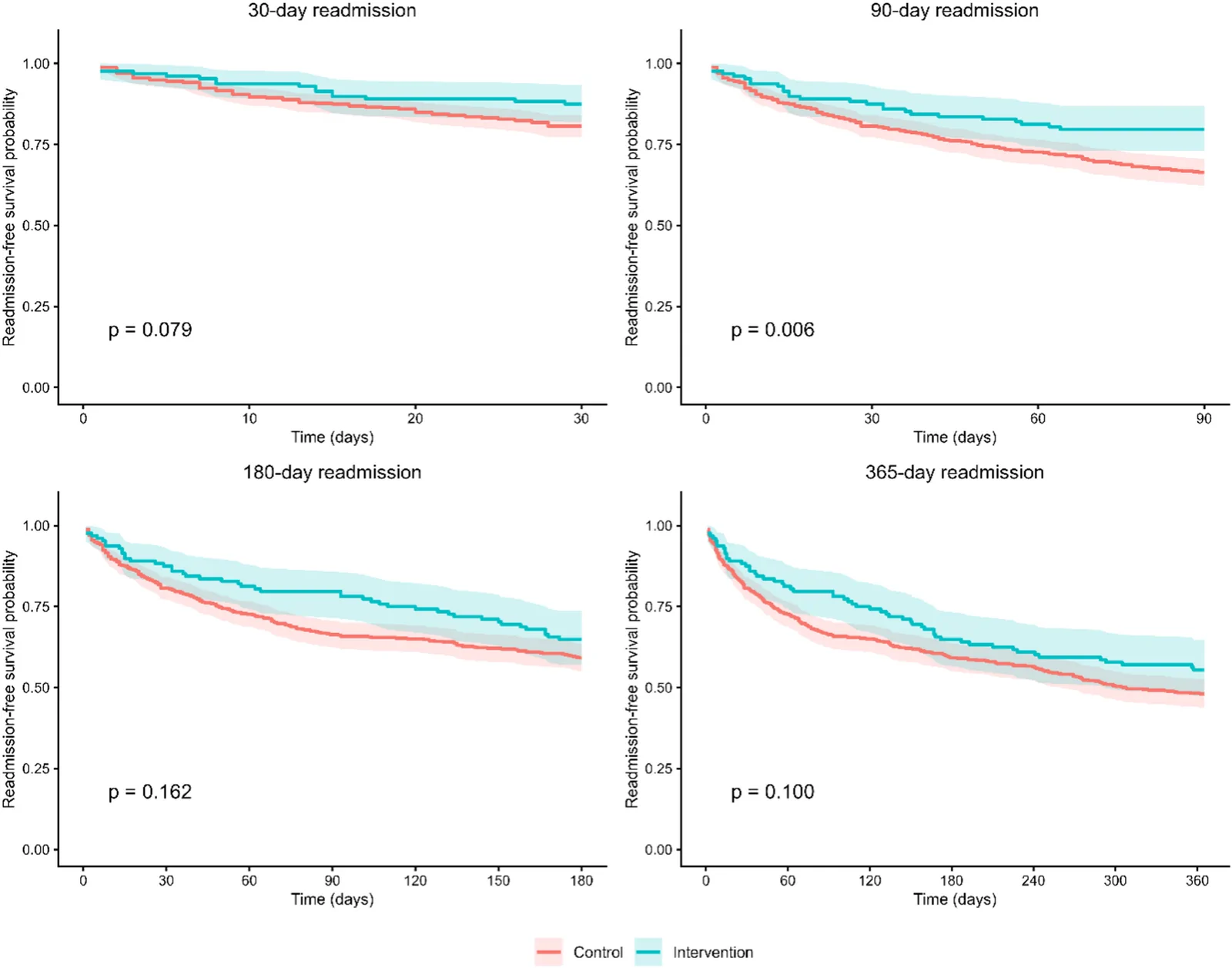
Kaplan–Meier estimates of readmission-free survival through 30, 90, 180 and 365 days following index hospital discharge. Death before readmission was treated as a censoring event

Kaplan–Meier estimates of readmission-free survival were higher in the intervention group, with the largest observed separation at 90 days (Fig. 4). At 90 days, readmission-free survival was 79.7% in the intervention group and 66.4% among controls (log-rank *p* = 0.006). Differences at 30, 180 and 365 days were less pronounced (log-rank *p* = 0.079, 0.162 and 0.100, respectively). Because several follow-up horizons were evaluated, the 90-day result was interpreted as exploratory.

### Competing risk analysis

When death before readmission was treated as a competing event, the cumulative incidence of first hospital readmission was lower in the intervention group at each evaluated follow-up horizon (Table 5). At 30 days, cumulative incidence was 12.5% (95% CI 6.8–18.3) in the intervention group and 19.3% (95% CI 15.9–22.8) among controls (Gray’s *p* = 0.076). The largest observed difference occurred at 90 days, when cumulative incidence was 20.3% (95% CI 13.3–27.3) in the intervention group compared with 33.6% (95% CI 29.5–37.7) in controls (Gray’s *p* = 0.005). At 180 days, cumulative incidence was 35.2% versus 40.8% (*p* = 0.157), and at 365 days it was 44.5% versus 52.0% (*p* = 0.096).

Fine–Gray models yielded a 90-day subdistribution hazard ratio (sHR) of 0.57 (95% CI 0.37–0.86; *p* = 0.008). At 30, 180 and 365 days, the corresponding sHRs were 0.63 (95% CI 0.36–1.08), 0.80 (95% CI 0.59–1.08) and 0.79 (95% CI 0.59–1.05), respectively. The corresponding cause-specific Cox estimates were similar in direction (Table 4). At 365 days, the cause-specific HR was 0.75 (95% CI 0.57–0.99), whereas the Fine–Gray sHR was 0.79 (95% CI 0.59–1.05), reflecting the different estimands addressed by the two approaches.

**Table 5:** Cumulative incidence and Fine–Gray analyses of first hospital readmission with death as a competing event

Time point	Intervention CIF % (95% CI)	Control CIF % (95% CI)	ARD, pp	Gray’s *p*-value	Fine–Gray sHR (95% CI)	*p*-value
30 days	12.50 (6.75–18.25)	19.34 (15.91–22.76)	6.84	0.076	0.63 (0.36–1.08)	0.091
90 days	20.31 (13.31–27.31)	33.59 (29.50–37.69)	13.28	0.005	0.57 (0.37–0.86)	0.008
180 days	35.16 (26.84–43.47)	40.82 (36.56–45.08)	5.66	0.157	0.80 (0.59–1.08)	0.137
365 days	44.53 (35.87–53.19)	51.95 (47.62–56.29)	7.42	0.096	0.79 (0.59–1.05)	0.098

ARD, absolute risk difference; CIF, cumulative incidence function; CI, confidence interval; pp, percentage points; sHR, subdistribution hazard ratio. ARD was calculated as the cumulative incidence in the control group minus that in the intervention group. Death before first readmission was treated as a competing event. Gray’s test was used to compare cumulative incidence functions. Fine–Gray models used robust standard errors clustered by propensity-score matched subclass. Controls were the reference group

## Discussion

In this retrospective propensity score-matched cohort study of patients aged 75 years or older, the *Safe homecoming* intervention was associated with a lower incidence of first hospital readmission at 90 days. The crude cumulative readmission proportion was 20.3% among intervention patients compared with 33.6% among matched controls. This association was observed consistently in the competing-risk analysis (Fine–Gray sHR 0.57, 95% CI 0.37–0.86) and in the cause-specific Cox model (HR 0.55, 95% CI 0.36–0.84). At 30 and 180 days, estimates were also below 1 but were less precise, while at 365 days the Fine–Gray estimate was 0.79 (95% CI 0.59–1.05). Mortality hazard ratios were below 1 at the evaluated follow-up horizons, but all confidence intervals included the null value, and the present study therefore does not provide robust evidence of a mortality effect.

The 90-day finding should nevertheless be interpreted cautiously. Several outcomes and follow-up horizons were evaluated without adjustment for multiplicity, and 90 days was not specified as a unique confirmatory endpoint. The observed association may represent a time-limited effect of early transitional care, but chance cannot be excluded. Consequently, the 90-day result should be considered exploratory and requires confirmation in prospectively designed studies.

Our findings align with previous research on transitional care [26, 27], which has similarly demonstrated inconsistent significance across key outcomes. For instance, only six out of 21 randomised controlled trials reported significant reductions in hospital readmissions, and just two out of 13 showed significant effects on mortality [26].

*Safe homecoming* provided its most intensive support during approximately the first two weeks following discharge. It is clinically plausible that early medication review, rehabilitation, coordinated care planning and recognition of deterioration during this vulnerable transitional period could influence subsequent healthcare utilisation beyond the active intervention period. Previous studies have also reported short-term effects of transitional-care interventions [9, 28–32]. However, the present study was not designed to determine why the largest statistical separation occurred specifically at 90 days, and 90 days should not be regarded as a mechanistically defined threshold. The temporal pattern may also reflect sampling variability. This is consistent with findings from several previous studies on transitional care [9, 28–31], which have predominantly reported short-term benefits in reducing hospital readmissions and mortality rates. Especially interventions initiated immediately after discharge contributed to improved health outcomes and reduced readmission rates [9, 28–31]. Additionally, findings from a multicentre trial have demonstrated that an intervention extending beyond 2 weeks may reduce emergency department visits, hospital readmissions and mortality [32]. These effects appear to be primarily attributable to enhanced opportunities for rehabilitation and self-management [32]. This aligns with the present intervention, where early rehabilitation was prioritised immediately after the patient returned home. However, our study did not evaluate the individual components of the intervention, nor did it explore the underlying reasons for readmission; instead, the focus has been on the comprehensive approach of the intervention, which is in line with other studies [26, 33] advocating a comprehensive approach to enhance post-discharge care.

Furthermore, tailoring interventions to contextual factors appears essential [26, 27]. A potential factor that may have influenced the results is the use of short -term municipal care following hospital discharge. In the Nordic healthcare context, short-term care is generally provided to older patients with substantial care or rehabilitation needs who may have difficulties returning directly home [34]. The *Safe homecoming* intervention may in some cases have enabled patients to be discharged directly home rather than to a short-term care facility. While short-term care provides more extensive support, it also involves an additional care transition. In contrast, *Safe homecoming* provided interprofessional, coordinated home-based care. Furthermore, physician and physiotherapist involvements are often more limited in standard short-term care settings, whereas the intervention included close collaboration with both general practitioner and physiotherapist as parts of the team. This difference in medical and rehabilitative follow-up should be considered when interpreting potential differences on outcomes. In addition, as data on short-term care placements where not available, the extent to which such placements influenced the observed outcomes remains uncertain. When comparing our results with similar studies conducted in Denmark [12, 35], a country with a health care system comparable to Sweden’s, mortality outcomes likewise failed to reach significance. However, one of the Danish studies [35] did report a significant reduction in readmission rates. In that study, the municipality-based intervention resembled standard care in Örebro county whereas their hospital-based intervention shared core components with the present *Safe homecoming* intervention, including structured discharge planning and multidisciplinary follow-up with active engagement of patients and their relatives. Importantly, in *Safe homecoming* the multidisciplinary component is expanded to prioritise interprofessional teamwork.

In our study, the 30-day crude readmission proportion was 12.5% in the intervention group and 19.3% among controls. Hansen et al. [35] reported readmission rates of 17.7% and 21.5% in their hospital-based and municipality-based intervention arms, respectively, while Rasmussen et al. [12] reported similar rates between intervention and control groups. These numerical comparisons provide clinical context but should not be interpreted as evidence that one intervention was more effective than another because eligibility criteria, case mix, intervention components, follow-up procedures and healthcare organisation differed between studies.

The direction of several estimates was compatible with a potentially lower risk among intervention patients; however, point estimates alone do not establish intervention effectiveness. The observational design, relatively small intervention sample and width of several confidence intervals require cautious interpretation. In particular, the 90-day association should be considered exploratory because multiple outcomes and follow-up horizons were evaluated without adjustment for multiplicity.

The non-randomised design is an important limitation of this study. Propensity score matching was used to improve comparability between the intervention and control groups with respect to measured baseline characteristics; however, matching cannot reproduce randomisation or eliminate unmeasured confounding. Selection into *Safe homecoming* occurred in routine clinical practice following professional assessment and may therefore have depended on characteristics not available in the study data, including functional status, frailty, cognition, rehabilitation potential, home environment, motivation and informal caregiver support. These factors could influence both intervention allocation and subsequent outcomes.

Controls were drawn from public primary health care centres across Örebro County rather than exclusively from Kumla. Consequently, unmeasured differences in healthcare organisation, municipal services, socioeconomic conditions and access to home care may also have contributed to the observed associations. Previous healthcare utilisation was not included in the final propensity-score model and may represent an additional source of residual confounding.

The lack of randomisation in our study design increases susceptibility to bias. To reduce the risk of selection bias, we strived to create comparable groups. Initially, different matching strategies were evaluated and used to compare the *Safe homecoming* intervention with standard care (see Supplementary Fig. 1 and Table 1, Additional file 2). Drawing controls from public primary health care centres across Örebro County enabled matching with up to four controls per intervention patient, increasing statistical precision. However, residual confounding due to unmeasured variation in health care organisations, municipal services, socioeconomic conditions, and health care utilisation prior to baseline may have influenced the observed outcomes. A further limitation of propensity score matching is its reliance on model assumptions, which may not fully eliminate hidden confounding factors [36]. Additionally, as this retrospective study is based on data from administrative and medical records, a methodological concern is the restricted data available for analysis. We were unable to include known confounders such as socioeconomic status, functional status, dependency in daily activities, frailty, cognitive function and access to informal care, which may have influenced the outcomes. Data on municipal short-term care placements were unavailable. This is an important limitation because the *Safe homecoming* intervention may in some cases have enabled patients to return directly home when short-term institutional care might otherwise have been considered. Differences in post-discharge care pathways between intervention patients and controls could therefore have influenced subsequent hospital utilisation and mortality and cannot be separated from the observed intervention association. The implementation of the new discharge legislation may have improved post-discharge care planning among controls, although such effects were likely modest during the study period and would, if anything, be expected to favour the control group.

Data were reviewed to exclude palliative care as a confounder at baseline, and no ICD-10 code Z51.5 indicating such care were found. These patients are most often recognised through care arrangement codes, which were not available in our dataset. This may have led to underreporting of patients with palliative care needs. Although administrative and medical records may be subject to coding inaccuracies and do not capture all clinically relevant variables, mortality and hospital readmission are routinely recorded outcomes and are therefore likely to be captured with a high degree of completeness and accuracy.

Despite these limitations, the study has notable strengths. The use of data from administrative records enhances the reliability of findings compared with self-reported data. Among the 640 patients included (128 intervention patients and 512 controls), there was no loss to follow-up. Although part of the data collection was conducted during the early phase of the COVID-19 pandemic, the impact of the pandemic appears to have been minimal. Only 15 controls were enrolled after week 12 of 2020, when the outbreak began to affect Sweden [37], and no ICD-10 codes for COVID-19 were recorded in our dataset. Even though undetected cases cannot be excluded, a misclassification is unlikely to have essentially affected the outcomes.

Strengths include complete follow-up for mortality and hospital readmission in the matched cohort, the use of administrative and medical-record data, propensity-score matching to address measured baseline differences and complementary time-to-event analyses accounting for death as a competing event. The consistency between the corrected 90-day cumulative-incidence, Fine–Gray and cause-specific Cox estimates support the internal coherence of the revised analyses. Although individual socioeconomic data were unavailable, the intervention municipality is socioeconomically representative of Örebro County, and both the intervention and control group included urban and rural communities. Nevertheless, these analytical methods cannot eliminate residual confounding inherent in the observational design. The 90-day association should nevertheless be interpreted cautiously given the limited intervention sample, observational design and evaluation of multiple follow-up horizons. Therefore, further studies with larger cohorts, preferably randomized controlled studies, are warranted to assess whether the observed lack of significance may be attributed to insufficient statistical power, particularly in the context of competing risk scenarios and across diverse health care settings. Future research should also aim to explore key components of the *Safe homecoming* intervention, including its optimal duration and adherence among frail subgroups. Further evaluation is needed to clarify the roles of interprofessional collaboration, medication reviews and informal caregiver involvement. Incorporating patient-reported outcomes, functional and cognitive assessments and experiences of patients and caregivers would offer deeper insight into the intervention’s effectiveness and perceived value. Additionally, health economic analyses are essential to assess its cost-effectiveness.

## Conclusions

In this retrospective propensity score-matched cohort study, the *Safe homecoming* intervention was associated with a lower risk of first hospital readmission at 90 days compared with matched controls. This association was observed in both cause-specific and competing-risk analyses. Mortality analyses did not show a statistically significant association, while evidence for differences in readmission at other follow-up horizons was less conclusive. Given the observational design, potential residual confounding, small intervention sample and evaluation of multiple follow-up horizons, the findings should be considered exploratory. Larger prospectively designed studies are needed to determine whether the observed 90-day association is reproducible and to assess the generalisability of this model of transitional care.

## Supplementary information

Below is the link to the electronic supplementary material.

Supplementary Material 1

Supplementary Material 2

## Data Availability

The datasets used and/or analysed during the current study are available from the corresponding author (UW) on reasonable request.

## Abbreviations

ARD: absolute risk difference
ATC: Anatomical Therapeutic Chemical
CCI: Charlson Comorbidity Index
CCIw: weighted Charlson Comorbidity Index
CI: confidence interval
CIF: cumulative incidence function
CIP: coordinated individual planning
HR: hazard ratio
ICD-10: International Classification of Diseases, 10th revision
N/A: Not applicable
PHC: primary health care
PP: percentages points
SD: standard deviation
sHR: subdistribution hazard ratio
SMD: standardised mean difference
