# Supplementary material for: Safe homecoming intervention after hospital discharge: a retrospective cohort study of mortality and readmission among patients 75 years or older in Sweden
Source: BMC Geriatr. 2026 Oct 6;26:1280. doi: 10.1186/s12877-026-08367-w (PMC13654218; doi:10.1186/s12877-026-08367-w)
Supplement: Supplementary file 1 [file 12877_2026_8367_MOESM1_ESM.docx]

Additional file 1

Contents

**Supplementary Table 1.** Covariates included in propensity score matching

**Supplementary Figure 1.** Love Plot Standardized mean differences before vs after propensity score matching

**Supplementary Table 2.** Top 10 first diagnoses at baseline by ICD-10 codes by cohort

**Supplementary Table 3.** Top 10 first ICD-10 chapters reflecting organ system or clinical speciality involved by cohort

**Supplementary Table 1.** Covariates included in Propensity Score Matching

| **Covariates in Propensity Score Matching** | **Values and categories** |
| --- | --- |
| Age | Plus/minus 2 years tolerance ≥75 |
| Sex | Male/Female |
| CCIw calculated the year prior baseline | 0;1-2;3-5;>5 (points) |
| Unique ATC-codes one day before baseline | 0-4;5-9;≥ 10 (n) |
| At baseline length of hospital stay in days | 1-6; 7-14; >15 |
| Main ICD-10 code category registered at baseline | All codes included in the CCI-script; All codes prefixed by S- or T-; All remaining codes (excluded CCI-script and prefixed by S-or T-). |

ATC, Anatomical Therapeutic Chemical classification of medications; CCIw, Weighted Charlson Comorbidity Index; ICD-10, International Classification of Diseases, 10th revision

**Supplementary Figure 1.** Love Plot: Standardised mean differences before versus after propensity score matching.

atc_cat, total unique ATC-codes categorised 0-4;5-9;≥ 10 (n) one day prior admission to hospital at baseline; cciw_cat, Charlson comorbidity index weighted year prior admission to hospital categorisation into 0; 1-2; 3-5; >5; diag_cat_ST_code, all ICD-10 codes initiated with S- and T- at baseline ; diag_cat_special_list, all ICD-10 codes excluded those enrolled in cciw and codes initiated with S- and T- at baseline; stay_cat_7-14, categorisation length of hospital stay 7-14 days at baseline; stay_cat_>15, length of hospital stay >15 days at baseline.

**Supplementary Table 2.** Top 10 first diagnoses at baseline by ICD-10 codes by cohort

| Interventions | | | |
| --- | --- | --- | --- |
| Rank ICD-10 codes n Percent | | | |
| 1 | S720 | 7 | 5.5% |
| 2 | I639 | 6 | 4.7% |
| 3 | S4220 | 5 | 3.9% |
| 4 | Z750 | 5 | 3.9% |
| 5 | J189 | 4 | 3.1% |
| 6 | N179 | 4 | 3.1% |
| 7 | S7200 | 4 | 3.1% |
| 8 | C209 | 3 | 2.3% |
| 9 | I509 | 3 | 2.3% |
| 10 | M485 | 3 | 2.3% |

| Controls | | | |
| --- | --- | --- | --- |
| Rank ICD-10 codes n Percent | | | |
| 1 | I639 | 23 | 4.5% |
| 2 | S7200 | 17 | 3.3% |
| 3 | S7210 | 17 | 3.3% |
| 4 | I509 | 16 | 3.1% |
| 5 | J189 | 16 | 3.1% |
| 6 | F039 | 8 | 1.6% |
| 7 | M549 | 8 | 1.6% |
| 8 | N109 | 7 | 1.4% |
| 9 | A469 | 6 | 1.2% |
| 10 | L039 | 6 | 1.2% |

ICD-10, International Classification of Diseases, 10th Revision; n, numbers of ICD-10 codes; S7200, fracture of neck of femur; I639, cerebral infarction; S4220, fracture of shaft of femur; Z750, medical services not elsewhere classified; J189, pneumonia, unspecified organism; acute kidney failure, unspecified; C209, malignant neoplasm of rectum, unspecified; I509, heart failure, unspecified; M485, collapsed vertebra, not elsewhere classified; S7210, fracture of trochanteric section of femur; F039, unspecified dementia; M549, dorsalgia, unspecified; N109, acute tubulo-interstitial nephritis, unspecified; A469, erysipelas, unspecified; L039, cellulitis, unspecified

**Supplementary Table 3.** Top 10 first ICD-10 chapters reflecting organ system or clinical speciality involved by cohort

| Interventions | | | |
| --- | --- | --- | --- |
| Rank Chapters n Percent | | | |
| 1 | S | 31 | 24.2% |
| 2 | I | 20 | 15.6% |
| 3 | C | 14 | 10.9% |
| 4 | J | 11 | 8.6% |
| 5 | R | 10 | 7.8% |
| 6 | N | 9 | 7.0% |
| 7 | Z | 9 | 7.0% |
| 8 | M | 7 | 5.5% |
| 9 | A | 3 | 2.3% |
| 10 | B | 3 | 2.3% |

| Controls | | | |
| --- | --- | --- | --- |
| Rank Chapters n Percent | | | |
| 1 | S | 91 | 17.8% |
| 2 | I | 87 | 17.0% |
| 3 | M | 53 | 10.4% |
| 4 | J | 47 | 9.2% |
| 5 | R | 43 | 8.4% |
| 6 | K | 36 | 7.0% |
| 7 | C | 30 | 5.9% |
| 8 | A | 21 | 4.1% |
| 9 | L | 17 | 3.3% |
| 10 | N | 17 | 3.3% |

ICD-10, International Classification of Diseases, 10th Revision; n, numbers of ICD-10 codes; S, Injuries to specific body regions; I, diseases of the circulatory system; C, neoplasm; diseases of the respiratory system; R, symptoms, signs and abnormal clinical/lab findings; N, diseases of the genitourinary system; Z, factors influencing health status and contact with health services; M, diseases of the musculoskeletal system and connective tissue; A-B, certain infectious and parasitic diseases; K, diseases of the digestive system; L, diseases of the skin and subcutaneous tissue
