# Supplementary material for: Safe homecoming intervention after hospital discharge: a retrospective cohort study of mortality and readmission among patients 75 years or older in Sweden
Source: BMC Geriatr. 2026 Oct 6;26:1280. doi: 10.1186/s12877-026-08367-w (PMC13654218; doi:10.1186/s12877-026-08367-w)
Supplement: Supplementary file 2 [file 12877_2026_8367_MOESM2_ESM.docx]

Additional file 2

Contents

**Supplementary Figure 1.** Flowchart of selection and matching process Charlson Comorbidity index and Morbidity classification

**Supplementary Tabel 1.** Descriptive statistics Charlson Comorbidity index and Morbidity classification

**Supplementary Figure 1.** Flowchart of selection and matching process Charlson Comorbidity index and Morbidity classification

CCIw, weighted Charlson comorbidity index; *,Morbidity classification based on age, sex, number of medications and days spent in hospital one-year prior inclusion.

**Supplementary Tabel 1.** Descriptive statistics Charlson Comorbidity index and Morbidity classification

| **Variable** | **Intervention patients CCIw file (n=132)** | **Controls CCIw file (n=517)** | **P-value** | **Intervention patients morbidity file (n=142)** | **Controls morbidity file (n=594)** | **P-value** |
| --- | --- | --- | --- | --- | --- | --- |
| Age, mean (SD)* | 84.56 (6.02) | 85.33 (5.66) | .137* | 84.82 (5.9) | 84.39 (5.7) | .492* |
| Sex male/female, %* | 40.9 / 59.1 | 42.7 / 57.3 | .663* | 42.6 / 57.4 | 41.1 / 58.9 | .749* |
| CCIw at inclusion,mean (SD) | 1.6 (1.7) | 1.32 (1.6) | .075 | N/A | N/A | N/A |
| CCIw at inclusion,min-max (median) | 0-8 (1) | 0-8 (1) | .075 | N/A | N/A | N/A |
| **CCIw categories at inclusion*** |  |  | .071* |  |  | N/A |
| CCIw categorie at inclusion, 0 (%) | 32.1 | 38.5 |  | N/A | N/A | N/A |
| CCIw categorie at inclusion, 1-2, (%) | 46.6 | 46.6 |  | N/A | N/A | N/A |
| CCIw categorie at inclusion, 3-4 (%) | 12.2 | 8.5 |  | N/A | N/A | N/A |
| CCIw categorie at inclusion, >5 (%) | 9.2 | 6.4 |  | N/A | N/A | N/A |
| **ICD-10 codes a year prior inclusion enrolled in CCIw** |  |  |  |  |  |  |
| ICD-10 code Myocardial infarction, % | 11.4 | 11.8 | .251 | 10.6 | 10.3 | .897 |
| ICD-10 code Congestive heart failure, % | 18.9 | 12.4 | .007 | 18.4 | 13.5 | .131 |
| ICD-10 code Peripheral vascular disease, % | 3.8 | 3.9 | .865 | 3.5 | 4.7 | .547 |
| ICD-10 code Cerebrovascular disease, % | 9.8 | 10.6 | .078 | 9.9 | 11 | .686 |
| ICD-10 code Chronic obstructive pulmonary disease, % | 8.3 | 7.5 | .789 | 8.5 | 6.2 | .329 |
| ICD-10 code Chronic other pulmonary disease, % | 4.5 | 6.6 | .035 | 4.3 | 5.4 | .586 |
| ICD-10 code Rheumatic disease, % | 7.6 | 5.2 | .005 | 7.1 | 4 | .121 |
| ICD-10 code Dementia, % | 6.1 | 7.9 | .005 | 5.7 | 7.9 | .364 |
| ICD-10 code Hemiplegia, % | 0 | 0.8 | .011 | 0 | 0 | N/A |
| ICD-10 code Diabetes without chronic complications, % | 0 | 0.4 | .475 | 0 | 0.2 | .626 |
| ICD-10 code Diabetes with chronic complications, % | 4.5 | 3.7 | .911 | 5 | 4.4 | .762 |
| ICD-10 code Renal disease, % | 9.1 | 5.6 | <.001 | 8.5 | 6.1 | .290 |
| ICD-10 code Liver disease, % | 0 | 0.6 | .381 | 0 | 0.3 | .491 |
| ICD-10 code Peptic ulcer disease, % | 0.8 | 1 | .791 | 0.7 | 1.3 | .536 |
| ICD-10 code Malignancy, % | 21.2 | 13.9 | <.001 | 21.3 | 15.3 | .087 |
| ICD-10 code Metatstatic cancer, % | 0.8 | 1 | .791 | 0.7 | 1.3 | .536 |
| ICD-10 code Aids, % | 0 | 0 | N/A | 0 | 0 | N/A |
| **Hospital admission** |  |  |  |  |  |  |
| Admissions one year prior inclusion, mean (SD)* | 1.64 (1.09) | 1.52 (1.04) | .137 | 0.6 (1.1) | 0.6 (0.9) | .750* |
| Admissions one year prior inclusion, min-max (median)* | 1-7 (1) | 1-10 (1) | .137 | 0-6 (0) | 0-6 (0) | .750* |
| Length of stay in days one year prior inclusion, mean (SD)* | 15,31 (11,3) | 9.54 (11.3) | <.001 | 15.09 (11.09) | 9.88 (11.77) | <.001* |
| Length of stay in days one year prior inclusion, min-max (median)* | 3-64 (11.5) | 0-81 (5) | <.001 | 3-64 (11) | 0-172 (6) | <.001* |
| Length of stay in days at inclusion, mean (SD) | 12.4 (8.8) | 6.2 (7.4) | <.001 | 12.33 (8.7) | 6.64 (9.1) | <.001 |
| Length of stay in days at inclusion, min-max (median) | 2-52 (10) | 0-63 (3) | <.001 | 1-52 (10) | 0-162 (4) | <.001 |

ATC, anatomical therapeutic chemical classification of medications; CCIw, weighted Charlson comorbidity index; ICD-10, international classification of diseases, 10th revision; N/A, not applicable *, variables included in matching.
